# TcMYB29a, an ABA-Responsive R2R3-MYB Transcriptional Factor, Upregulates Taxol Biosynthesis in *Taxus chinensis*

**DOI:** 10.3389/fpls.2022.804593

**Published:** 2022-03-04

**Authors:** Xiaoying Cao, Lingxia Xu, Ludan Li, Wen Wan, Jihong Jiang

**Affiliations:** Key Laboratory of Biotechnology for Medicinal Plants of Jiangsu Province, School of Life Sciences, Jiangsu Normal University, Xuzhou, China

**Keywords:** transcription factor, R2R3-MYB, taxol biosynthesis, *Taxus chinensis*, taxane 5α-hydroxylase

## Abstract

Paclitaxel (Taxol), a highly modified diterpene agent mainly obtained from *Taxus* species, is the most widely used anticancer drug. Abscisic acid (ABA) is a well-known stress hormone that plays important roles in the secondary metabolism of plants, and it can also induce the accumulation of taxol in *Taxus* cell suspension cultures. However, the mechanism behind the regulation of taxol biosynthesis by ABA remains largely unknown. In previous research, a R2R3 MYB transcription factor (TF) TcMYB29a was observed to show a significant correlation with taxol biosynthesis, indicative of its potential role in the taxol biosynthesis. In this study, the TcMYB29a encoded by its gene was further characterized. An expression pattern analysis revealed that *TcMYB29a* was highly expressed in the needles and roots. Overexpression of *TcMYB29a* in *Taxus chinensis* cell suspension cultures led to an increased accumulation of taxol, and upregulated expression of taxol-biosynthesis-related genes, including the taxadiene synthase (TS) gene, the taxane 5α-hydroxylase (T5OH) gene, and the 3′-N-debenzoyl-2′-deoxytaxol-N-benzoyltransferase (DBTNBT) gene as compared to the controls. Chromatin immunoprecipitation (ChIP) assays, yeast one-hybrid (Y1H) assays, electrophoretic mobility shift assays (EMSAs), and dual-luciferase reporter assays verified that TcMYB29a could bind and activate the promoter of *TcT5OH.* Promoter sequence analysis of *TcMYB29a* revealed that its promoter containing an AERB site from -313 to -319 was a crucial ABA-responsive element. Subsequently, the ABA treatment assay showed that *TcMYB29a* was strongly upregulated at 6 h after ABA pretreatment. Furthermore, *TcMYB29a* was strongly suppressed at 3 h after the methyl jasmonate (MeJA) treatment and was depressed to the platform at 12 h. Taken together, these results reveal that TcMYB29a is an activator that improves the accumulation of taxol in *Taxus chinensis* cells through an ABA-medicated signaling pathway which is different from JA-medicated signaling pathways for the accumulation of taxol. These findings provide new insights into the potential regulatory roles of MYBs on the expression of taxol biosynthetic genes in *Taxus*.

## Introduction

**FIGURE 1:**
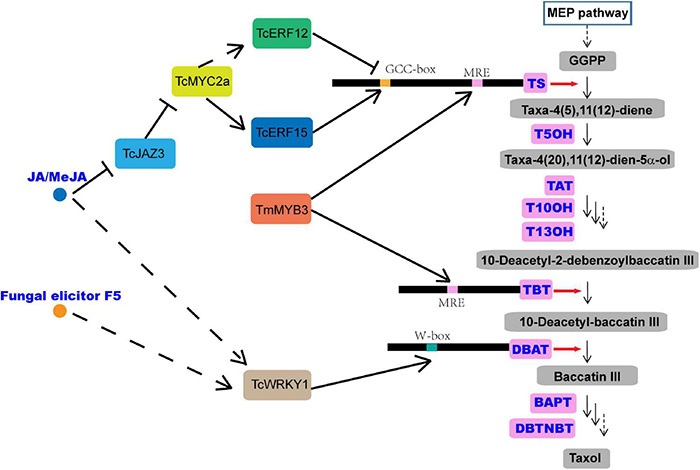
Overview of the taxol biosynthesis pathway. MEP, 2-C-methyl-d-erythritol 4-phosphate; TS, taxadiene synthase; GGPP, geranylgeranyl diphosphate; T5OH, taxadiene-5α-hydroxylase; TAT, taxadien-5α-ol O-acetyltransferase; T10OH, taxane 10β-hydroxylase; T13OH, taxane 13α-hydroxylase; TBT, taxane 2α-O-benzoyltransferase; DBAT, 10-deacetylbaccatin-III-10-β-O-acetyltransferase; BAPT, baccatin III-3-amino-3-phenylpropanoyltransferase; DBTNBT, 3′-N-debenzoyltaxol-N-benzoyltransferase; MRE, MYB recognition element; JAZ, jasmonate ZIM-domain protein.

*Taxus chinensis* is an endangered and economically valuable medicinal woody species of the genus, *Taxus*. Its bark can produce taxol (generic name: paclitaxel), which is one of the most effective anticancer drugs derived from natural sources and is widely used in the treatment of various solid tumors, such as breast, ovarian, and lung cancer and Kaposi’s sarcoma (Wani et al., 1971; Gallego-Jara et al., 2020). Taxol is a taxane diterpene, and its biosynthesis mainly needs two metabolic pathways, the diterpenoid pathway and the phenylpropanoid pathway. The former provides the main taxane carbon skeleton, baccatin III, and the latter offers the phenylisoserine side chain (Croteau et al., 2006; Ssmsa and Mnr, 2020). The highly complex taxol biosynthesis pathway involves more than 20 enzymes, which catalyze at least 19 steps of reactions and convert the universal diterpenoid precursor, geranylgeranyl diphosphate (GGPP), into taxol (Ssmsa and Mnr, 2020; Figure 1). The first step of taxol biosynthesis, a cyclization of GGPP into taxa-4(5),11(12)-diene, is catalyzed by the taxadiene synthase (TS), which is a slow-starter and a rate-limiting enzyme for the provision of the key intermediate 10-deacetylbaccatin III (10-DAB) (Figure 1). Taxane 5α-hydroxylase (T5OH), a cytochrome P450 enzyme, catalyzes the first oxygenation step of taxol biosynthesis, in which taxa-4(5),11(12)-diene is transformed into taxa-4(5),11(12)-diene-5α-ol (Koepp et al., 1995; Figure 1), and 3′-N-debenzoyl-2′-deoxytaxol-N-benzoyltransferase (DBTNBT), an important enzyme, is involved in the formation of the functional taxol molecule, which converts the 3′-N-debenzoyltaxol into taxol (Figure 1).

Jasmonic acid (JA) and its derivative, methyl jasmonate (MeJA), among the most important stress hormones, have been evaluated as the most effective inducers of taxol biosynthesis in *Taxus* cell cultures (Li et al., 2013; Lenka et al., 2015; Zhang et al., 2015, 2018). Recently, transcription factors (TFs), including WRKY, MYC, basic helix-loop-helix (bHLH), ethylene responsive factor (ERF), and MYB, have been isolated, characterized, and identified as regulators of taxol biosynthesis in *Taxus* (Li et al., 2013; Lenka et al., 2015; Zhang et al., 2015, 2018), and most of them have been verified to be JA-responsive. TcWRKY1, a JA-responsive WRKY transcription factor isolated from *Taxus chinensis*, participates in the regulation of taxol biosynthesis by directly activating the expression of 10-deacetylbaccatin-III-10-b-O-acetyltransferase (DBAT) gene (Li et al., 2013). Another two JA-responsive WRKY TFs, TcWRKY8 and TcWRKY47 in *T. chinensis*, significantly increase the expression levels of several taxol biosynthetic genes, including *DBAT*, *T5OH*, and *TcERF15* (Zhang et al., 2018). The MYC family members, which are the core TFs in the JA signaling pathway, are also found to act in the regulation of taxol biosynthesis (Lenka et al., 2015). The interaction between jasmonate ZIM-domain (JAZ) proteins and MYC proteins is also detected in *Taxus media* in a similar mode of action as in model plants. In a model, JAZs can bind with MYCs and inhibit their transcriptional activities (Cui et al., 2019; Figure 1). In *Taxus cuspidata*, three JA-inducted MYC TFs, TcJAMYC1, TcJAMYC2, and TcJAMYC4, are verified to be involved in the negative regulation of the expression of taxol biosynthetic genes in *Taxus cuspidata* (Lenka et al., 2015). Another study showed that TcMYC2a may regulate the expression of *TS*, *TAT*, *DBTNBT*, *T13OH*, and *T5OH* directly or indirectly through ERF12 and ERF15 that are dependent on the JA signaling pathway in *Taxus chinensis* (Li et al., 2013; Zhang et al., 2018; Figure 1).

The MYB protein family, defined by the myb domain, constitutes one of the largest groups of TFs described in the plant kingdom, and it acts as a key factor in the regulatory networks controlling various processes in plant growth and development and in response to the biotic and abiotic stresses (Yanhui et al., 2006; Dubos et al., 2010). The repeat motif (R) of the myb domain usually comprises about 52 amino acids and forms three α-helices, in which the second and third helices of each domain build a helix-turn-helix (HTH) fold and are involved in specifically binding to their target DNA sequences (Ogata et al., 1996; Dubos et al., 2010; Baldoni et al., 2015; Hou et al., 2018). Based on the number of myb repeats, the MYB proteins are classified into four subfamilies, including R1R2R3R1/2-MYBs (4R-MYBs), R1R2R3-MYBs (3R-MYBs), R2R3-MYBs, and MYBs (1R-MYBs and MYB-related) in plants (Dubos et al., 2010). Among them, the R2R3-MYB subfamily contains the most abundant MYB proteins and includes more than 100 members in different species, such as *Arabidopsis thaliana* and *Populus trichocarpa* (Wilkins et al., 2009; Dubos et al., 2010; Hou et al., 2018). The R2R3-MYB TFs have a modular structure with a highly conserved myb domain for DNA-binding at the N-terminal (Baldoni et al., 2015) and a transcription activation or repression region usually located in the C-terminus of the proteins. Amino acid motifs in the C-terminal of R2R3-MYB proteins are rich in acidic amino acid residues that are responsible for the diverse regulatory functions of the TFs (Araki et al., 2004; Daniel et al., 2004; Morse et al., 2009). Numerous R2R3-MYB proteins have been characterized and have been found to play central roles in the secondary metabolism of plants (Martin and Paz-Ares, 1997). For instance, R2R3-MYBs are well known for their regulatory role in the pathways of anthocyanin biosynthesis, a specific branch of the phenylpropanoid pathway, by forming a triad with bHLH and WD40 TFs (Wang et al., 2021b). The overexpression of SmMYB9b, an R2R3-MYB TF isolated from *Salvia miltiorrhiza*, could increase the accumulation of tanshinone in *S. miltiorrhiza* (Zhang J. et al., 2017). Also, SmMYB98, an R2R3-MYB TF, isolated from this medicinal herb, could simultaneously promote the biosynthesis of tanshinone and salvianolic acid in *S. miltiorrhiza* (Hao et al., 2020). Two MYB TFs, BpMYB21 and BpMYB61 in birch, regulate triterpenoid biosynthesis (Yin et al., 2020). Furthermore, TmMYB3, an R2R3-MYB TF, has been found to be involved in taxol biosynthesis in *Taxus media*, by activating the expression of *TmTBT* and *TmTS* (Yu et al., 2020). Although previous studies have indicated that the MYBs are involved in the regulation of taxol biosynthesis (Li et al., 2012; Wang et al., 2019; Yu et al., 2020); to date, only a few *MYB* genes have been identified in the *Taxus* spp.

Except for JA, the ABA is considered as another stress hormone for its important roles in response to various biotic and abiotic stresses in plants (Wang et al., 2021a,b). Although ABA has also been evaluated and found to induce taxol biosynthesis in *Taxus* cell cultures (Zhang and Fevereiro, 2010), its function in the regulation of the taxol biosynthesis is largely unknown. In our current research (Cao et al., 2022), the fermentation broth of an endophytic fungus isolated from *T. chinensis* could significantly promote taxol accumulation in *T. chinensis* needles, with a 3.26-fold increase compared to the control. TF-encoding genes, such as MYBs, ERFs, and bHLH, were detected as differentially expressed genes after KL27-FB treatment. In these TFs, an *MYB* gene showed a high expression after treatment with the fungal elicitor, and the overexpression of the *MYB* gene could significantly improve the accumulation of taxol in the cells of *Taxus callus*. Promoter sequence analysis showed that its promoter contains an ABA-responsive element but not a JA-responsive element, suggesting its potential role in the regulation of taxol biosynthesis by ABA. A homolog search and a phylogenic analysis identified the MYB as a typical R2R3-MYB TF that shows high similarity with R2R3-MYB transcription factor 29 (QHG11457.1) (TcMYB29) (Hu et al., 2020) and was thus renamed as TcMYB29a in this study. Although Hu et al. (2020) performed a comprehensive analysis of TcMYB29, the function of TcMYB29 in taxol biosynthesis in *Taxus* was not studied deep. A series of experiments were then performed to determine the function of TcMYB29a in the taxol biosynthesis and to explain the regulation system.

## Materials and Methods

### Plant Materials and Transformation of *Taxus chinensis* Calli

The *Taxus chinensis* plants used were grown in pots containing soil in the greenhouse of the Jiangsu Normal University, Xuzhou, China at 22°C with 50% relative humidity. The needles, stem epidermis, phloem, xylem, and roots were collected from 5-year-old *T. chinensis* seedlings. Specimens of the tissue were collected from three plants. All samples were frozen in liquid nitrogen and stored at –80°C until RNA extraction.

*T. chinensis* calli used for the genetic transformation were cultured in aseptic bottles containing a solid medium of modified B5 (Lee et al., 2010), which contains 4 g/L of plant gel and 20 g/L of surcose. The calli were cultured at 25°C in the dark, and the wild-type and transgenic calli were subcultured at 2-week intervals on the modified B5 solid medium.

Recombinant plasmids were transformed into *T. chinensis* calli using the *Agrobacterium*-medicated transformation (Zhang et al., 2015). After infection for 30 min, the calli were transferred into the modified B5 solid medium in the dark for 2 days at 25°C. The cocultured calli were rinsed with a fresh liquid medium containing 50 μg/mL of kanamycin and 300 μg/mL of cefotaxime, then transferred into a fresh solid medium containing 50 μg/mL of kanamycin and 300 μg/mL of cefotaxime, and cultured in the dark at 25°C.

### RNA Isolation and Quantitative Real-Time PCR

The RNA isolation was performed by the EASYspin plant RNA extraction kit (Aidlab Bio., China) according to the instructions of the manufacturer. The quality and quantity of the RNA were determined by a NanoDrop2000c spectrophotometer (Thermo Fisher Scientific, United States), and the RNA integrity was identified by electrophoresis on 1.0% of agarose gels. The complementary DNA (cDNA) was synthesized from 1.0 μg of total RNA using the HiScript II Q RT SuperMix by a quantitative PCR (qPCR) kit with gDNase (Vazyme, China) according to the protocols of the manufacturer. To determine the expression levels of *TcMYB29a* in different tissues or treatments, a quantitative real-time PCR (qRT-PCR) analysis was carried out using the ABI StepOnePlus Real-time PCR systems (Thermo Fisher Scientific, United States). One microliter of synthesized cDNA (diluted at 1:5) was used as the template for the qRT-PCR. Specific primers of each gene are listed in Supplementary Table 1. The *TcGAPDH* was selected as a reference gene (Zhang et al., 2020). Amplification cycles included 30 s at 95°C, followed by 40 cycles at 95°C for 15 s and 60°C for 30 s. Each measurement was performed with three biological replicates. Data were analyzed by using the 2^–ΔΔCT^ method.

### Construction of Overexpression and RNAi Vectors

The coding region of the *TcMYB29a* was taken from the transcriptome datasets of *T. chinensis* from previous research. Specific primers (Supplementary Table 1) were designed to amplify the *TcMYB29a* DNA segment from the cDNA of *T. chinensis* using the following PCR parameters: initial denaturation at 95°C for 3 min, 30 cycles of denaturation at 95°C for 15 s, annealing at 58°C for 30 s, extension at 72°C for 2 min, and final extension at 72°C for 10 min. The PCR products were subcloned into the pHB-GFP vector with *Bam*HI and *Xba*I restriction sites to form pHB-TcMYB29a-OE. The sequence (1,584 bp) of the *TcMYB29a* gene was used to construct an intron-spliced hairpin RNA (RNAi construct) to inhibit the expression of *TcMYB29a*. The amplified fragment was inserted into the pHB vector by reverse orientation to construct the RNAi vector, pHB-TcMYB29a-RNAi. The vectors, including pHB-TcMYB29a-OE, pHB-TcMYB29a-RNAi, and pHB (set as a control), were transformed into the *T. chinensis* calli. Each experiment was conducted using more than three biological replicates.

### Sequence Analysis of TcMYB29a

The molecular weight and theoretical isoelectric point of TcMYB29a were computed using the compute pI/Mw tool on the ExPASy server.^1^ A BLAST search (BlastX)^2^ was used for a homology search from the SWISS-PROT protein database. The TcMYB29a protein was predicted on PlantTFDB.^3^ Classification of the myb-like DNA binding domain was predicted using the online software, HMMER3.3.^4^ Multiple sequence alignments of the full-length MYB proteins were performed using clustalW in default settings. A phylogenetic tree was described using the MEGA Version 11 adopting the Neighbor-Joining algorithm, and the reliability of the branching pattern was tested with 1,000 bootstrap repetitions.

### Subcellular Localization of TcMYB29a in *Taxus* and the Epidermis Cells of Tobacco

The recombinant vector, 35S:GFP-TcMYB29a, was transformed into the *Agrobacterium tumefaciens* strain, GV3101, for plant transformation. *T. chinensis* calli were transformed as described above, and the transgenic *Taxus* calli were then placed on modified B5 plates at 25°C and in the dark for 2 days. Transient expression in tobacco leaves was assessed according to a published method (Zhou et al., 2018). The leaf epidermis of tobacco was peeled to make a temporary squash. After incubation with a phosphate-buffered saline containing 4′,6′-diamidino-2-phenylindole (DAPI), the temporary squash and *Taxus* calli were then observed under a Leica Fluorescence Microscope at 10 × 20 (Leica Microsystems, Wetzlar, GmBH).

### *Cis*-Element Analysis of Promoter Sequences

Genome DNA was extracted by a Plant Genomic DNA extraction Kit (Aidlab Biotech, China) and used as a template. The specific primers for *TcT5OH*, *TcTS*, *TcBAPT*, and *TcDBTNBT* promoters were designed based on the known sequences of the cDNA (accepted from the transcriptome datasets in *T. chinensis* in previous research) (Supplementary Table 1) and four general primers provided by the Genome Walking Kit (Takara). Genome walking PCR was performed by applying the Genome Walking Kit, and the PCR products were cloned, sequenced, and aligned with the designed partial sequences of the open reading frame of *TcT5OH*, *TcTS*, *TcBAPT*, and *TcDBTNBT* genes, respectively, to decide the 5′-flanking region. The promoter sequence of *TcMYB29a* was achieved from the *T. chinensis* genome sequence downloaded from the NCBI. Then, the online database PLACE^5^ and PlantCARE^6^ were used to identify the *cis*-acting elements of the promoters.

### Chromatin Immunoprecipitation PCR

Chromatin immunoprecipitation (ChIP) was conducted with the transgenic *Taxus* calli harboring 35S:GFP-TcMYB29a using the method reported in the literature (Meng et al., 2018). The analysis of ChIP DNA products was performed by qRT-PCR using primers that were synthesized to amplify the DNA fragments in the promoter regions of *TcTS*, *TcT5OH*, *TcBAPT*, and *TcDBTNBT* genes. The primer sequences for R-1 and R-2, R-3 and R-4, R-5 to R-7, and R-8 and R-9 were used for the amplification of DNA fragments in the promoters of *TcTS*, *TcT5OH*, *TcBAPT*, and *TcDBTNBT*, respectively. Primer sequences TcTSqF and TcTSqR (CDS-1), TcT5OHqF and TcT5OHqR (CDS-2), TcBAPTqF and TcBAPTqR (CDS-3), and TcDBTNBTqF and TcDBTNBTqR (CDS-4) were used for amplifying the regions of coding sequences (CDSs) in *TcTS*, *TcT5OH*, *TcBAPT*, and *TcDBTNBT*, respectively, and were set as an internal control (Supplementary Table 1; Wang and Lindås, 2018). These experiments were repeated more than three times.

### Yeast One-Hybrid Assays

To confirm the interaction between TcMYB29a and the promoters of *TcBAPT* and *TcT5OH* genes, yeast one-hybrid (Y1H) assays were conducted using the Matchmaker^®^ Gold Yeast One-Hybrid Library Screening System (Clontech, CA, United States) according to the instructions of the manufacturer. The full-length cDNA segment of *TcMYB29a* was cloned into the *Eco*RI-*Xho*I sites of the GAL4 activation vector (pGADT7-Rec) to form pAD-TcMYB29a. The promoter fragment containing the putative MYB recognition elements (MREs), B1 and B2 of the *TcT5OH* promoter and P5 of *TcBAPT*, were separately amplified from the genome DNA of *T. chinensis* with primers (Supplementary Table 1). The DNA segments containing three repeats of B1 binding sites and their mutants were synthesized by Sangon Biotech Co. (Shanghai). Then, these fragments and oligos were cloned into the *Sac*I-*Sal*I sites of the pABAi vector to form pY1-ABAi, pY2-ABAi, p3B1-ABAi, p3mB1-ABAi, and pY3-ABAi using a ClonExpress II One step Cloning Kit (Vazyme, China). Following the protocols of the manufacturer, the vectors were cotransformed into the yeast strain, Y1HGold, according to LiAc conversion protocols. The transformed yeast cells were diluted to an OD_600nm_ of 0.005, dropped onto a selective medium containing a synthetic dextrose (SD) without Ura and Leu (SD/-Ura/-Leu), and SD/-Ura/-Leu with 50 ng/mL of ABA (SD/-Ura/-Leu + 50 ng/mL of ABA), 100 ng/mL of ABA (SD/-Ura/-Leu + 100 ng/mL of ABA), and 150 ng/mL of ABA (SD/-Ura/-Leu + 150 ng/mL of ABA), and incubated at 30°C for 48 h.

### Electrophoretic Mobility Shift Assays

The coding region of *TcMYB29a* was subcloned into the pGEX-4T-1 vector to form GST-TcMYB29a, in which a GST-tag was fused into the N-terminal of the TcMYB29a. The resulting plasmid was transformed into *Escherichia coli* Rosetta (DE3). The 3′-end biotin B1 probe corresponding to the B1 site was prepared (Sangon Bio, China) (Supplementary Table 1). Both the mutant probes containing two mutated nucleotides and the B1 probe were without a biotin label and were set as a competitor probe. The electrophoretic mobility shift assays (EMSAs) were conducted using a LightShift Chemiluminescent EMSA Kit (Thermo Fisher Scientific, United States) according to the instructions of the manufacturer.

### Dual-Luciferase Reporter Assays

To further measure the regulation of *TcT5OH* expression by TcMYB29a protein, dual-luciferase reporter assays were performed. For transcription activity analysis, the coding region of *TcMYB29a* was cloned into the pHB-GFP vector with *Hin*dIII and *Pst*I restriction sites under the control of the 35S promoter as an effector (35Spro:TcMYB29a). The promoter sequence of *TcT5OH* and its mutant, *mTcT5OH*, were inserted into a pGreenII 0800-Luc vector and were then cotransformed with 35Spro:TcMYB29a or free pHB vector (35S:pro, set as a negative control) into *Nicotiana benthamiana* leaves, adopting an *Agrobacterium*-mediated method described previously (Zhou et al., 2018). After being cultivated in the dark for 6 h and under long-day conditions (16 h/8 h, day/night) for 36 h, the transformed leaves were sprayed with a D-luciferin sodium salt (Solarbio, Beijing, China) and then examined by adopting a Bio-Rad Gel Doc XR (Bio-Rad, United States). Each assay was carried out with three biological replicates. The sequences of the primers are listed in Supplementary Table 1.

### Liquid Chromatography-Mass Spectrometry Analysis of Taxanes

The taxanes, including 10-deacetylbaccatin III, baccatin III, and taxol were studied by liquid chromatography-mass spectrometry (LC-MS) as described previously (Kayan et al., 2021). In brief, transgenic *Taxus* calli were frozen in liquid nitrogen, freeze-dried, and ground to powder using a mortar; about 0.1 g powder was mixed with 3 mL of 100% methanol and was then ultrasonicated three times for 60 min each. After centrifugation at 5,000 rpm for 5 min, the supernatant liquor was collected and extracted three times with dichloromethane/water (1:1, v/v). The organic fraction was collected, dried in vacuum, resuspended in 1 mL of methanol, and filtered through a 0.45-μm of organic-phase filter. The column used in all the experiments was a Poroshell 120 EC-C18 (4.6 × 150 mm, 4 μm) column (Agilent Technologies, Cheadle, United Kingdom) and the temperature for the chromatographic separation was set to 45°C. About 10 μl of the sample was injected in each chromatographic run. Mobile phases, delivered at 1.0 mL/min, consisted of 0.1% of formic acid either in 2.0 mmol/L of ammonium acetate aqueous solution (mobile phase A) and acetonitrile (mobile phase B). The chromatographic gradient was set as follows: 50% B for 0–4 min followed by a gradient to 95% B for 2 min, an isocratic step at 95% B for 1 min and then a gradient of50% B for 0.1 min, and an isocratic step at 50% B for 2.9 min. Mass spectral data were attained in positive electrospray mode (ESI +) in the multi-reaction monitoring mode. Operating conditions were optimized as follows: spray voltage of 5,500 V, ion source temperature of 550°C, curtain gas pressure at 25 psi (172 kPa), Ion Source Gas 1 at 50 psi (345 kPa), Ion Source Gas 2 at 60 psi (414 kPa), and residence time of 100 ms.

### Methyl Jasmonate and Abscisic Acid Treatments

*In vitro* long-term subcultured *T. chinensis* calli were maintained on the liquid modified B5 medium for 2 days; then, 8 g of cells were suspended in 50 mL of fresh liquid modified B5 medium, and incubated at 25°C while being shaken at 100 rpm for 48 h in the dark. Next, for MeJA and ABA treatments, the final concentrations of 50 μmol/L of MeJA or 20 μmol/L of ABA and the same volume of ethanol (set as the control) were added to the liquid medium, respectively, incubated at 25°C, and shaken at 100 rpm on a rotary shaker. These samples were harvested and frozen in liquid nitrogen after MeJA treatment at 0, 0.5, 1, 3, 6, 12, and 24 h, or after the ABA treatment at 0, 6, 24, 48, and 72 h for gene expression analysis, respectively.

## Results

### Cloning and Basic Analysis of TcMYB29a

**FIGURE 2:**
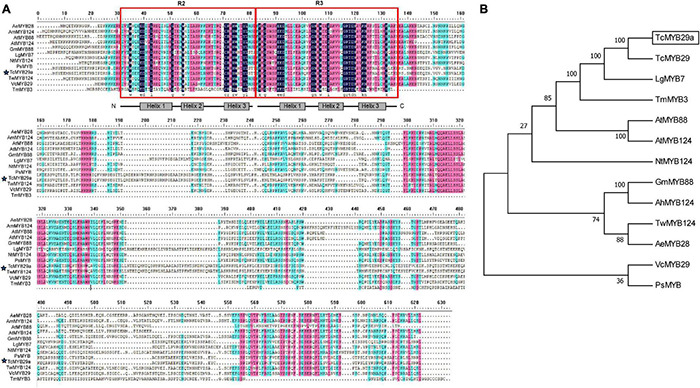
Amino acid sequence alignment and phylogenetic analysis of TcMYB29a. **(A)** Amino acid sequence alignment of TcMYB29a and other known R2R3-MYBs in plants. Conserved residues are highlighted in blue and partial conservation is shown in pink and green. The R2 and R3 domains are indicated with red boxes. The TcMYB29a studied in this study is indicated by blue stars. **(B)** The phylogenetic tree of TcMYB29a. Phylogenetic analysis of full-length TcMYB29a; TcMYB29a is framed by a solid box. The following GenBank accession numbers are used: *Arabidopsis thaliana* AtMYB88 (NP_565291.2), AtMYB124 (NP_001077534.1); *Taxus chinensis* TcMYB29 (QHG11457.1), *Larix gmelinii var. olgensis* LgMYB7 (QFG01315.1), *Nicotiana tomentosiformis* NtMYB124 (XP_009593376.1), *Glycine max* GmMYB88 (XP_003519765.1), *Arachis hypogaea* AhMYB124 (XP_025699147.1), *Vaccinium corymbosum* VcMYB29 (AYC35407.1), *Paeonia suffruticosa* PsMYB (QIG55701.1), *Tripterygium wilfordii* TwMYB124 (XP_038693483.1), and *Abelmoschus esculentus* AeMYB28 (QST87265.1). The amino acid sequence of *Taxus media*, TcMYB3, has been taken from previous research (Yu et al., 2020).

Based on the transcriptomes of *T. chinensis*, the full-length of the CDS of TcMYB29a was cloned. Sequence analysis indicated that *TcMYB29a* encodes a protein containing 527 amino acids with a predicted molecular weight of 60.18 kDa and a theoretical pI of 7.95. Multiple sequence alignments revealed that TcMYB29a contains two R motifs in the N-terminal of the amino acid sequences and belongs to the R2R3-MYB protein; there is no common repressor domain, such as EAR or TLLLFR motifs, identified in its C-terminus (Figure 2). BlastX hits analysis indicated that TcMYB29a shares the highest similarity with R2R3-MYB transcription factor 29 (QHG11457.1) in *T. c*hinensis (98.87%) (Hu et al., 2020). Phylogenetic analysis indicated a greater similarity among TcMYB29a, TcMYB29, and *Larix gmelinii var. olgensis* MYB3 (Figure 2). Hu et al. (2020) reported that TcMYB29, AtMYB88, and AtMYB124 were clustered in the S26 subgroup with counterparts in *Arabidopsis thaliana* and *T. chinensis.*

### TcMYB29a Localized in Nuclei

**FIGURE 3:**
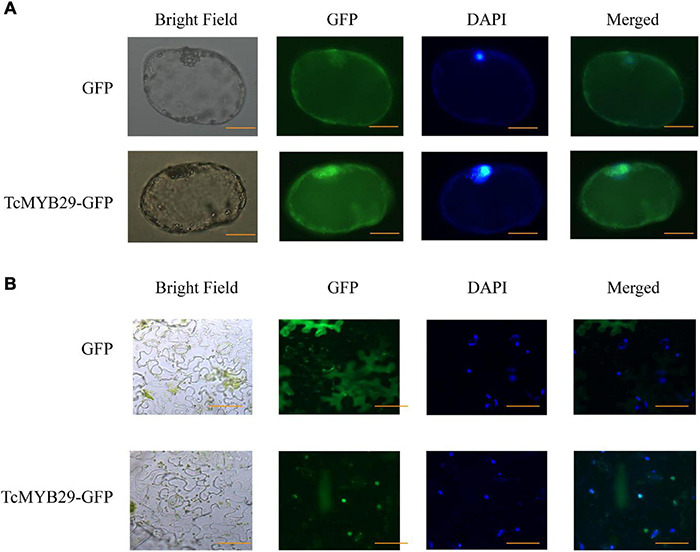
Subcellular localization of TcMYB29a in the **(A)** *Taxus* cells and **(B)** *Nicotiana tabacum*. The fused protein of TcMYB29a and GFP were transformed into *Taxus* cells. GFP fluorescence was observed 2 days after infection by laser scanning with 10 × 20 magnification. Photographs were taken in bright light and dark field for examining the GFP and diamidino-2-phenylindole (DAPI), respectively), and in combination (merged). Scale bar represents 40 μm.

The subcellular localization of TcMYB29a was performed *in vivo*. The location of TcMYB29a in plant cells was examined using GFP as a marker. In the epidemics cells of the tobacco leaf that transiently express GFP-TcMYB29a, the GFP signals were located in the nuclei. In the cells of *Taxus* callus that stably express GFP-TcMYB29a, the GFP signals were also observed in the nuclei and colocated with the nuclei marker dye, DAPI (Figure 3). However, when tobacco leaf epidemics cells and *Taxus* callus cells harboring the control vector carrying GFP alone were used as controls, the GFP was located in the cytoplasm and the nuclei in the tobacco leaf epidemics cells and *Taxus* callus cells (Figure 3). These results indicated that the GFP-TcMYB29a was localized in the nuclei (Figure 3), which is consistent with the prediction that TcMYB29a acts as a TF.

### Expression Pattern of *TcMYB29a*

**FIGURE 4:**
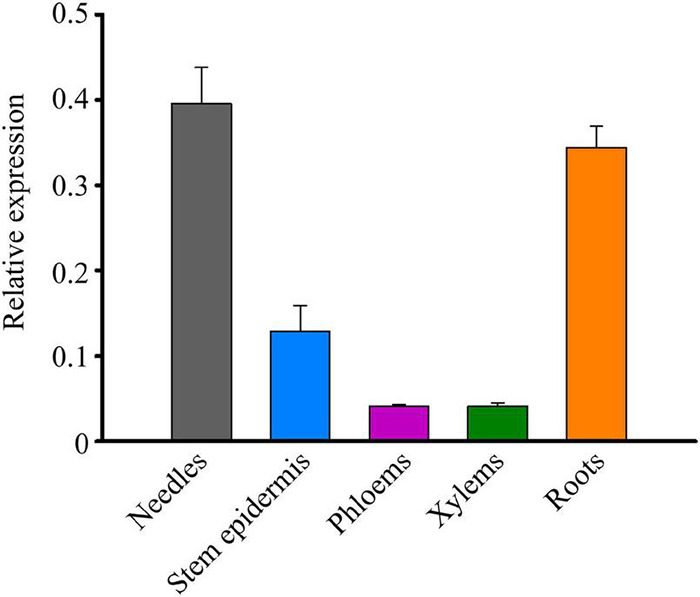
The expression patterns of *TcMYB29a* in the different tissues of *T. chinensis.* The expression of *TcMYB29a* was compared with that of *TcGAPDH*.

To probe the expression pattern of *TcMYB29a* during the growth and development of *T. chinensis*, the expression levels of *TcMYB29* in the needles, stem epidermis, phloem, xylem, and roots were tested using the qRT-PCR. The transcription of *TcMYB29a* was high in both the needles and roots, while low in both the phloem and xylem (Figure 4). Except in roots, the expression patterns of *TcMYB29a* in the needles, the phloem, and the xylem are consistent with the results of its most similar TcMYB29 in the findings of Hu et al. (2020).

### TcMYB29a Binds to the Promoter of *TcT5OH*

**FIGURE 5:**
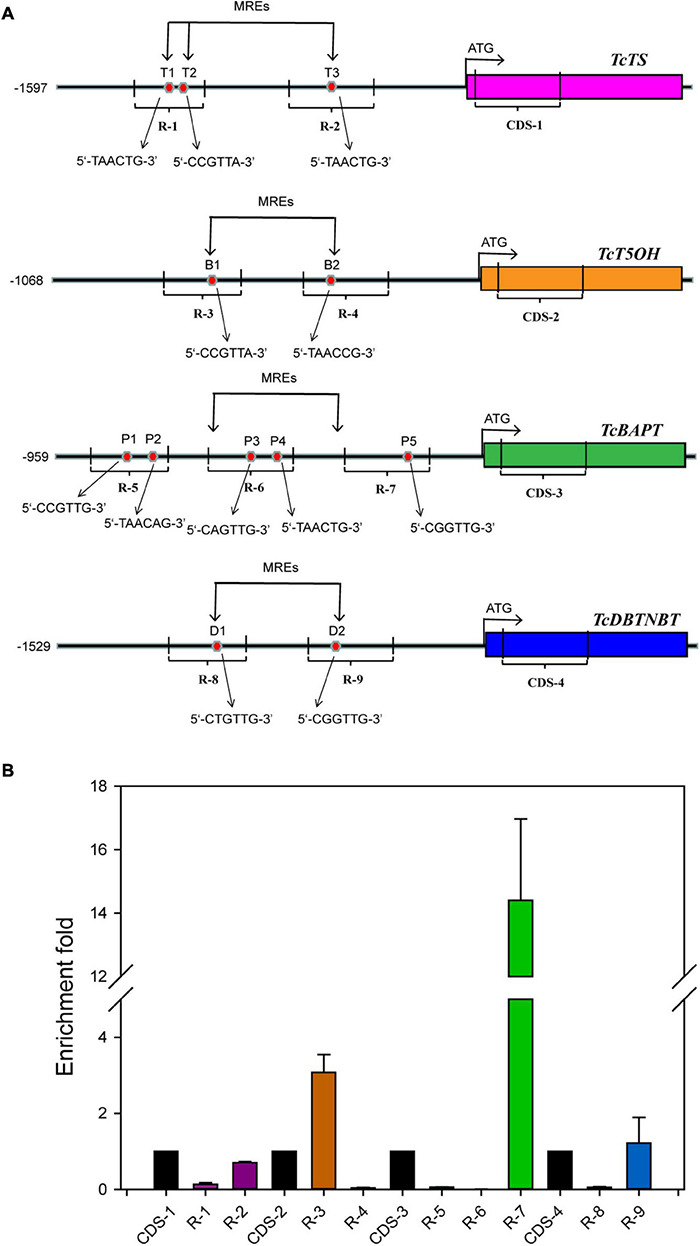
ChIP analysis. **(A)** Schematic of ChIP analysis on *TcTS*, *TcT5OH*, *TcBAPT*, and *TcDBTNBT* promoter locus and nine amplicons. **(B)** Enrichment of particles, *TcTS*, *TcT5OH*, *TcBAPT*, and *TcDBTNBT* promoter chromatin regions with the anti-GFP antibody in TcMYB29a-OE *Taxus* cells as detected by qPCR, respectively. Error bars represent SD for three independent experiments. R-1 to R-9 represent the promoter regions including T1 to T3, B1and B2, P1 to P5, and D1 and D2, respectively.

The role of *TcMYB29a* in the transcription of taxol biosynthesis-related genes was evaluated. Based on the information about the *T. chinensis* genome and genome walking approach, most promoter sequences of taxol biosynthesis-related genes were isolated. Results showed twelve putative MREs (5′-CNGTTR-3′): two MREs in the promoter of *TcT5OH* (-286 to-291 bp and from −488 to −493 bp), three MREs in the promoter of *TcTS* (from −1,346 to −1,341 bp, from −1,312 to −1,307 bp, and from −817 bp to −812 bp), five MREs in the promoter of *TcBAPT* (from −813 to −808 bp, from −787 to −782 bp, from −431 to −426, from −409 to −404, and from −188 to −183 bp), and two MREs in the promoter of *TcDBTNBT* (from −1,097 to −1,092 bp and from −681 to −676 bp) (Figure 5A), indicating that TcMYB29a may regulate the expression of *TcTS*, *TcT5OH*, *TcBAPT*, and *TcDBTNBT* by directly binding with their promoters. To test this possibility, the TcMYB29a over-expressed (OE)-*Taxus* calli were collected and used for ChIP analysis. As shown in Figure 5, the region R-3 of *TcT5OH* promoter and R-7 of *TcBAPT* promoter resulted in 3.23-fold and 14.41-fold enrichment compared to the CDS regions of *TcT5OH* and *TcBAPT* (CDS-2 and CDS-3), respectively. While R-4 in *TcT5OH* promoter, R-1 and R-2 in *TcTS* promoter, R-5 and R-6 in *TcBAPT* promoter, and R-8 and R-9 in *TcDBTNBT* promoter did not show enrichment compared with their controls. These results suggested that TcMYB29a may bind with the promoters of *TcT5OH* and *TcBAPT*.

**FIGURE 6:**
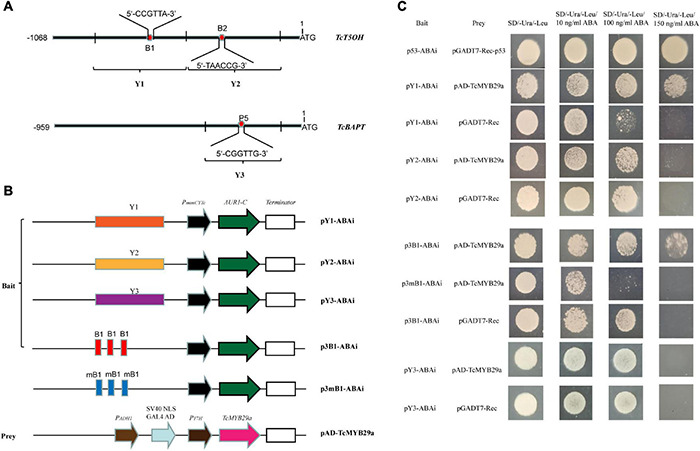
TcMYB29a could bind with MRE in yeast. **(A)** The sketch map of the promoters of *TcT5OH* and *TcBAPT* genes, the red and blue boxes indicate MRE, and B1, B2, and P5 show the putative MREs, respectively. Y1, Y2, and Y3 represent the promoter regions, including B1, B2, and P5, respectively. **(B)** Sketch map of the prey vector and bait vectors. mB1 indicates that the MRE was mutated as shown in Supplementary Table 1. **(C)** The binding capability tests of TcMYB29a and *cis*-acting elements in yeast. Related bait vectors with each *cis*-acting element were cotransformed with vacant pGADT7-Rec into the Y1HGold as the control.

Binding of TcMYB29a to *TcT5OH* and *TcBAPT* promoters was further validated by the Y1H assay. As shown in Figure 6, the yeast Y1HGold cell with pAD-TcMYB29a and bait vector pY1-ABAi grew well on an SD/-Ura/-Leu + 150 ng/mL of ABA. While the Y1HGold cells with pGADT7-Rec and bait vector, pY1-ABAi, with pAD-TcMYB29a and bait vector, pY2-ABAi, and with pGADT7-Rec and bait vector, pY2-ABAi were unable to grow when the concentration of ABA separately reached 150 ng/mL, indicating that TcMYB29a could bind with the Y1 sequence region of the promoter of the *TcT5OH* gene. However, the YIH analysis suggested that TcMYB29a did not bind to the *TcBAPT* promoter (Figure 6).

**FIGURE 7:**
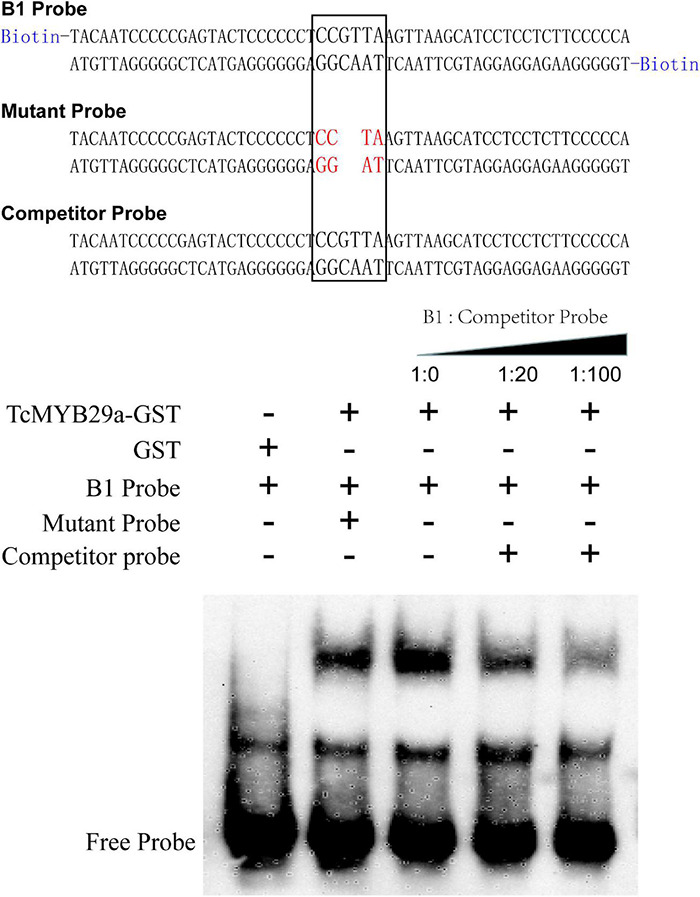
The electrophoretic mobility shift assay (EMSA) showing the interaction between TcMYB29a and the *TcT5OH* promoter.

Then, to further identify the direct binding site of the TcMYB29a with the *TcT5OH* promoter, treble MRE B1 was synthesized and used as the bait: Y1HGold cells containing the bait vector, p3B1-ABAi, and pAD-TcMYB29a grew well on an SD/-Ura/-Leu/150 ng/mL of ABA, while the Y1HGold cells containing the mutant B1 bait vector, p3mB1-ABAi and pAD-TcMYB29a, and p3B1-ABAi and pGADT7-Rec were unable to grow on the plates when the ABA concentration separately reached 100 ng/mL and 150 ng/mL, implying that TcMYB29a may directly bind with the B1 site in the promoter of the *TcT5OH* gene. Furthermore, EMSA was also used to assess the binding between TcMYB29a and the TcT5OH promoter *in vitro*. Based on the EMSA, the TcMYB29a binds to the B1 site in the *TcT5OH* promoter (Figure 7). Both *in vivo* and *in vitro* results expounded that TcMYB29a could bind with the MRE B1 in the promoter of *TcT5OH*, indicating that *TcT5OH* might be a downstream target of TcMYB2a.

### TcMYB29a Activates the Expression of *TcT5OH*

**FIGURE 8:**
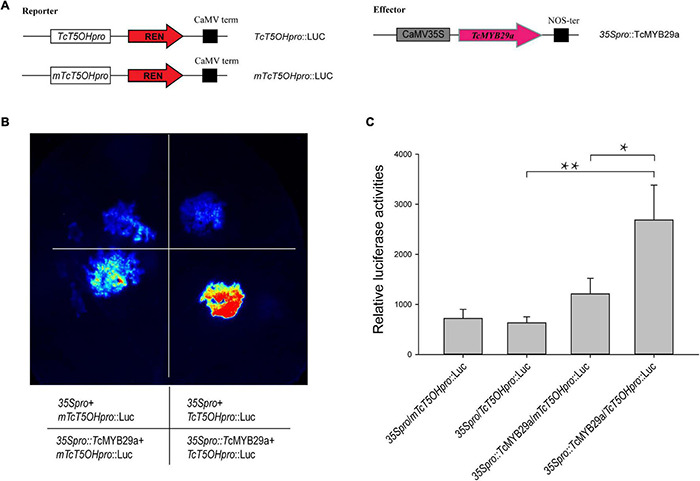
Transcriptional activation ability of TcMYB29a. **(A)** Schematic view of the plasmid combinations of dual-LUC reporters and effector. The promoter fragments of *TcT5OH* and its mutant, *mTcT5OHpro*, were cloned into the pGreenII 0800-LUC vector to generate the reporter constructs. The effector was generated by recombining the *TcMYB29a* gene into the pHB-GFP vector. LUC, firefly luciferase; REN, Renilla luciferase. **(B)** Effects of TcMYB29a on the promoter activity of *TcT5OH* as demonstrated by luciferase reporter assay. TcMYB29a significantly affected the promoter activity of *TcT5OH*. Deletion of two nucleotides in the sequence of the *mTcT5OH* promoter (the MRE B1 site in *TcT5OH* promoter CCGTTA was present in the mutant form as TACC). **(C)** Quantitative analysis of luminescence intensity. Three biological replicates were performed. The *p*-values were evaluated using Student’s *t*-test. Stars indicate the level of significance, *0.01 < *p* < 0.05, and **p < 0.01.

Dual-luciferase reporter assays were performed to validate the effect of TcMYB29a on the promoter activity of *TcT5OH*. As shown in Figure 8, the cotransformation of TcT5OHpro:Luc and 35Spro:TcMYB29a exhibited significantly higher luciferase activities than the control. However, the cotransformation of 35Spro:TcMYB29a and mTcT5OHpro:Luc showed significantly lower luciferase activities compared to that of 35Spro:TcMYB29a and TcT5OHpro:Luc. These results suggested that TcMYB29a significantly influences the promoter activity of *TcT5OH*, and the MEB B1 is an important *cis*-acting element for the activity of the *TcT5OH* promoter by way of TcMYB29a (Figure 8).

### Overexpression of TcMYB29a Promotes Taxol Accumulation in *Taxus* Calli

**FIGURE 9:**
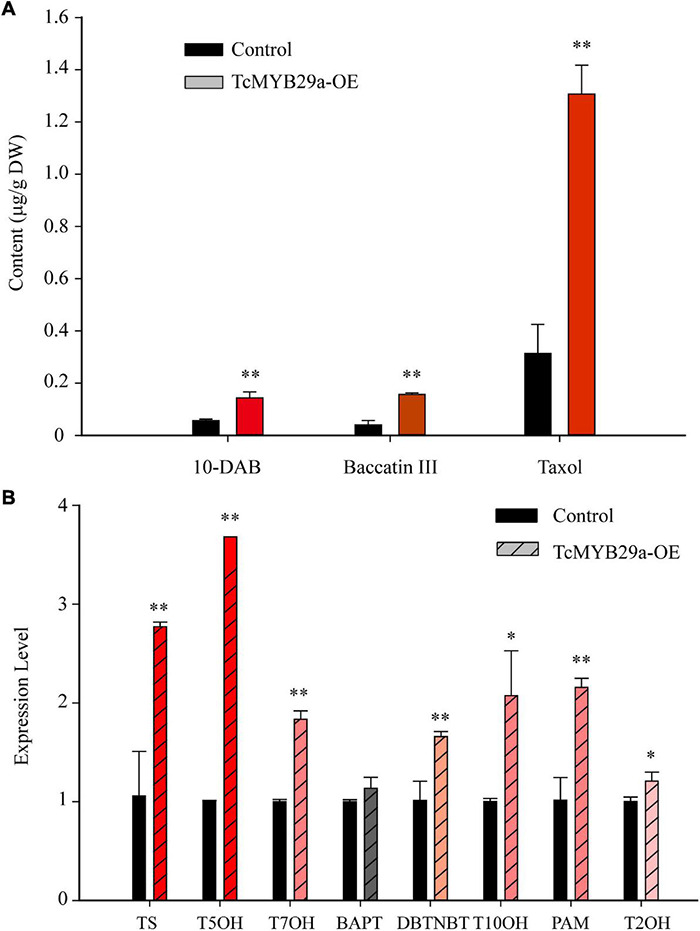
Taxane biosynthesis and taxol biosynthesis-related genes were upregulated in TcMYB29a-OE cells. **(A)** The liquid chromatography-mass spectrometry (LC-MS) quantified the contents of taxanes in the control and TcMYB29a-OE cells. **(B)** The qRT-PCR analysis of the expression of taxol biosynthesis-related genes in the control and in the TcMYB29a-OE cells. *TcGAPDH* was set as a control gene, and each experiment was conducted with three biological replicates. The *p*-values were evaluated using Student’s *t*-test. Stars indicate the level of significance, **p < 0.01, and *0.01 < *p* < 0.05. *TS*, taxadiene synthase; *T5OH*, taxadiene 5-alpha hydroxylase; *T7OH*, taxane 7-beta hydroxylase; *TcBAPT*, phenylpropanoyltransferase; *DBTNBT*, 3′-N-debenzoyl-2′-deoxytaxol-N-benzoyltransferase; *T10OH*, 5-alpha-taxadienol-10-beta-hydroxylase; *PAM*, phenylalanine aminomutase.

To corroborate the function of *TcMYB29a* in the process of taxol biosynthesis, 35S:GFP-TcMYB29a and its related empty plasmid 35S:GFP (set as a control) were introduced into *Taxus* calli. Three independent transgenic lines of both *TcMYB29a* and the control were chosen for taxane analysis using LC-MS. The contents of taxol and its main precursors (10-DAB and baccatin III) in the OE-calli were higher than those in the control samples. The contents of 10-DAB, baccatin III, and taxol separately rose by 238% from 0.06 ± 0.006 to 0.143 ± 0.31 μg/g of dry weight (DW), 400% from 0.04 ± 0.017 to 0.16 ± 0.006 μg/g of DW, and 419% from 0.313 ± 0.111 to 1.31 ± 0.111 μg/g of DW compared to the control group (*p* < 0.01) (Figure 9A and Supplementary Figure 2). However, RNAi interference showed no significant effect on the taxol biosynthesis in *T. chinensis* calli (Supplementary Figure 3) compared to the control, which may be due to the complex regulatory network for taxol biosynthesis.

Additionally, qRT-PCR analysis of taxol biosynthesis pathway genes showed that the expression of *TS*, *T50H*, *T7OH*, *DBTNBT*, *T10OH*, *T2OH*, and *PAM* were significantly upregulated in TcMYB29a-OE calli, while the expression of *BAPT* had no significant change (Figure 9B). These results suggested that TcMYB29a could activate most genes of the taxol biosynthesis pathway and significantly improved the taxol biosynthesis in *T. chinensis* calli.

### Abscisic Acid Improved *TcMYB29a* Gene Expression

**FIGURE 10:**
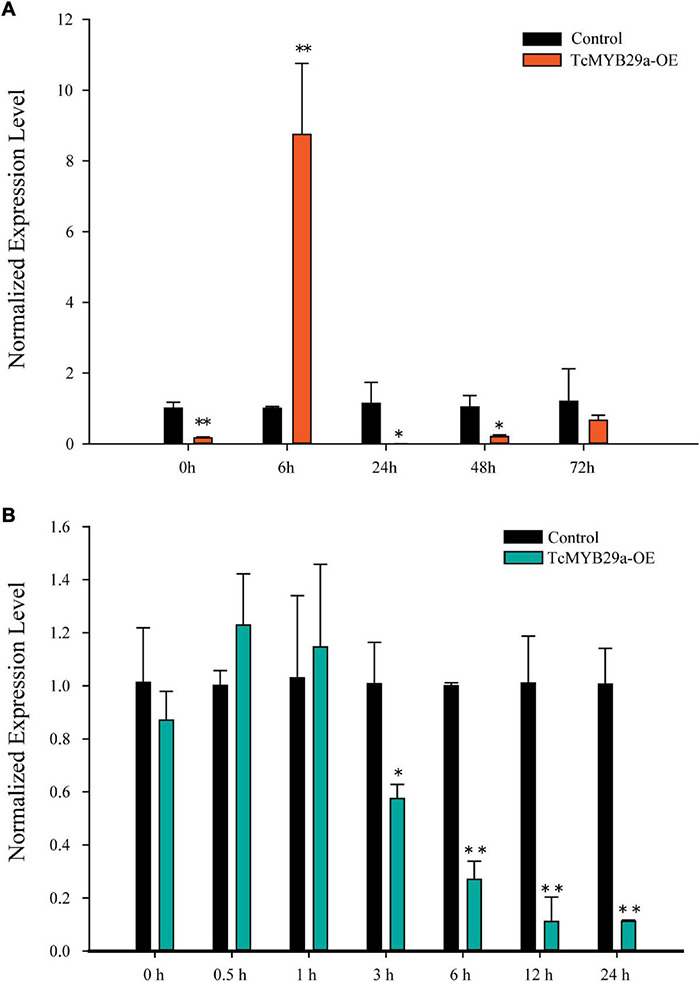
Expression patterns of *TcMYB29a* after ABA and MeJA treatments. The suspended *T. chinensis* cells were treated with 20 μmol/L of ABA **(A)** and 50 μM of MeJA **(B)**, respectively, with the solvent ethanol set as a control; the expression levels of *TcMYB29a* were detected at 0, 6, 24, 48, and 72 h after the ABA treatment, and at 0, 0.5, 1, 3, 6, 12, and 24 h after the MeJA treatment, respectively. The ordinate indicates the normalized expression levels compared to the sample with the added solvent. The *TcGAPDH* was set as a control gene; each experiment was conducted with three biological replicates. The *p*-values were evaluated using the Student’s *t*-test. Stars indicate the level of significance, *0.01 < *p* < 0.05, and ^**^p < 0.01.

Both ABA alone and ABA pretreatment of *Trollius yunnanensis* cell suspension cultures before hot stress-induction could significantly enhance taxol yield (Zhang and Fevereiro, 2010). The sequence analysis revealed that the *TcMYB29a* promoter contains a potential ABA regulatory element. To assess whether the expression of *TcMYB29a* is a response to ABA in *T. chinensis* cells, an RT-qPCR was conducted (Figure 10A). Six hours after the ABA treatment, the expression of *TcMYB29a* was significantly upregulated (8.75-fold compared to the control). However, the expression of *TcMYB29a* was downregulated at 24 h and returned to its initial level some 72 h after the ABA-treatment. In conclusion, *TcMYB29a* was an early response evincing the expression, having been improved by ABA signaling, thereby suggesting that TcMYB29a may participate in the regulation of taxol biosynthesis *via* an ABA-mediated pathway for improving the taxol biosynthesis (Figure 10A).

### Methyl Jasmonate Suppressed *TcMYB29a* Gene Expression

Methyl jasmonate is the most effective elicitor for taxol biosynthesis in *Taxus* suspended cells (Zhang M. et al., 2017). Most of the reported TFs involved in the taxol biosynthesis are JA-induced (Li et al., 2012, 2013; Lenka et al., 2015; Zhang et al., 2015, 2018). To explore the expression pattern of *TcMYB29a* in response to MeJA in *T. chinensis*, an RT-qPCR was performed (Figure 10B). In the initial stage of the MeJA treatment, the expression of *TcMYB29a* had only a very weak upregulation; however, after 3 h of the MeJA treatment, the expression of *TcMYB29a* was decreased significantly, remaining at 8.97-fold downregulation after 12 h. These results indicated that *TcMYB29a* had a later response to MeJA elicitor, and its expression was suppressed by MeJA signaling, thereby suggesting that TcMYB29a may participate in the regulation of taxol biosynthesis *via* a pathway that is different from the JA-mediated signaling pathway for improving the taxol biosynthesis.

## Discussion

Taxol, a microtubule-stabilizing drug widely used for treating various cancers, is a diterpenoid mainly isolated from the *Taxus* spp. However, the content of taxol in *Taxus* spp. is especially low and expensive to synthesize (Cragg et al., 1993; Wickremesinhe and Arteca, 1994). A deep understanding of the regulatory mechanism of the taxol biosynthesis pathway is helpful to improve the yield of taxol in *T. chinensis* by metabolic engineering (Wilson and Roberts, 2014).

**FIGURE 11:**
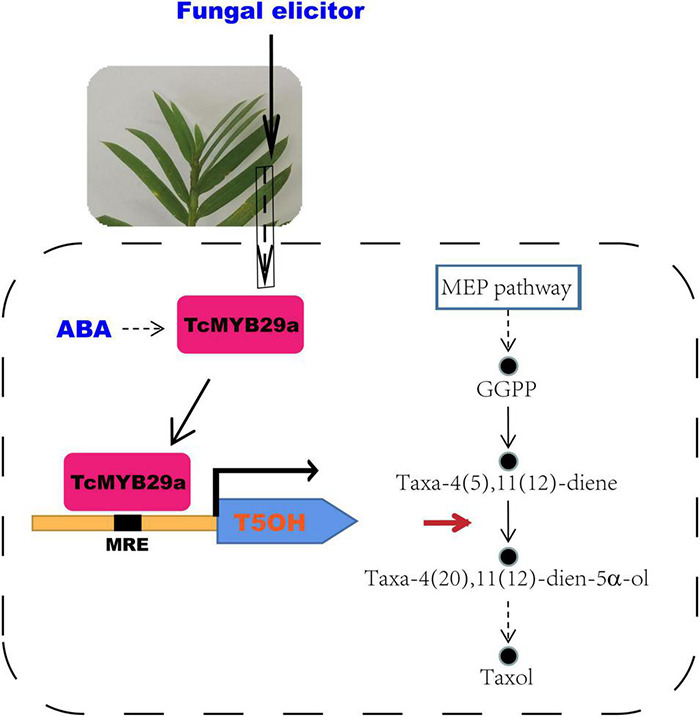
A model for the role of TcMYB29a in the taxol biosynthesis. GGPP, geranylgeranyl diphosphate; MEP, 2-C-methyl-D-erythritol 4-phosphate; MRE. MYB recognition element.

Transcription factors play important roles in the biosynthesis of secondary metabolites. In the *Taxus* species, several TFs, including WRKYs and ERFs in *T. chinensis*, MYCs in *T. cuspidata* and *T. chinensis*, and MYB in *T. media*, were proved as being involved in the taxol biosynthesis pathway (Li et al., 2013; Lenka et al., 2015; Zhang et al., 2015, 2018; Cui et al., 2019; Yu et al., 2021). Although MYB family genes have been characterized from different plants, little is known about its specific functions and mechanisms on the regulation of taxol biosynthesis in *Taxus* (yew). Several previous studies based on transcriptome sequencing and comparative analysis suggest that MYBs may be involved in regulating the taxol biosynthesis in *Taxus* (Li et al., 2012; Wang et al., 2019). However, the regulatory mechanism of those MYBs in the synthesis of taxol is not further explored. Recently, a phloem-specific R2R3-MYB TF, *TmMYB3*, was isolated from *T. media* and was confirmed to act as a transcriptional activator in the taxol biosynthesis, through binding directly to the promoters of *TmTBT* and *TmTS* genes (Yu et al., 2021). Also, according to previous research, an R2R3-MYB TF (named TcMYB29a) was isolated. The expression of *TcMYB29a* in the needles of *T. chinensis* was upregulated after an endophytic fungus elicitor treatment (Cao et al., 2022). This elicitor treatment improved the accumulation of taxol in *T. chinensis* needles, implying that the *TcMYB29a* gene may be involved in the regulation of taxol biosynthesis. Overexpression of *TcMYB29a* in *Taxus* cells could upregulate the expression of most taxol biosynthesis-related genes, especially the *TS* and *T5OH*. An LC-MS analysis also expounded that the contents of taxol and its main precursors, 10-DAB and baccatin III, increased in the cells of TcMYB29a-OE. Subsequently, Y1H, ChIP, EMSA, and LUC-assays further demonstrated that TcMYB29a can regulate the downstream gene expression by binding with the B1 site within the promoter region of *TcT5OH*. All these results indicated that TcMYB29a can directly and indirectly activate the expression of taxol biosynthesis-related genes and improved the taxol biosynthesis in the *Taxus* cells (Figure 11).

Overexpression of *TcMYB29a* has been found to be able to significantly upregulate the expression of *TS* and *T5OH* genes during the taxol biosynthesis in the *Taxus* cells. However, ChIP results showed that TcMYB29a was not bound with the predicted MREs in the *TcTS* promoter *in vivo*, implying that there may be unknown factors mediating the indirect regulation of TcMYB29a on the *TS* gene. TS, rather than T5OH, is a rate-limiting enzyme in the taxol biosynthesis, especially in the *Taxus* culture calli in which the accumulation of baccatin III and the *TS* gene expression were very low compared to their original levels in plants (Koksal et al., 2011; Ssmsa and Mnr, 2020); so, the increasing contents of taxol and its precursors (baccatin III and 10-DAB) in the cells of TcMYB29a-OE may be mainly due to the high expression of the *TS* gene. However, since *T5OH* was identified as a direct target of TcMYB29a, it is possible that the TcMYB29a regulator controls the taxol biosynthesis pathway at least partly by directly regulating the *T5OH* gene expression (Figure 11). Therefore, the factors that mediate the activation of TcMYB29a in the *TS* gene require further research.

Methyl jasmonate is confirmed to be one of the most effective inducers of taxol biosynthesis in *Taxus* suspended cell cultures (Zhang M. et al., 2017), except for the TmMYB3, which is not verified as to whether it was induced by MeJA; almost all reported TFs involved in the regulation of taxol biosynthesis are identified as having been induced by MeJA, such as TcWRKY (Li et al., 2012), MYC transcription factors, TcJAMYC1, TcJAMYC2, TcJAMYC4 (Lenka et al., 2015), TcMYC2a (Li et al., 2013; Zhang et al., 2018), TcERF12, and TcERF15 (Zhang et al., 2015). The MYC TFs are key regulators of the JA signaling pathway. This suggests that different members of the MYC family might perform different roles in the regulation of the taxol biosynthesis pathway (Lenka et al., 2015). For example, with MeJA elicitation, the transcript of *T5OH* was significantly repressed in the TcJAMYC2-OE *Taxus* cells but was not significantly regulated in the TcJAMYC1-OE and TcJAMYC4-OE *Taxus* cells (Lenka et al., 2015). In this study, the expression of *TcMYB29a* in *Taxus* callus cells was suppressed by MeJA. However, TcMYB29a is a positive regulator of *T5OH* gene in the taxol biosynthesis. Thus, further studies are needed to uncover the regulatory rules between MYC TFs and TcMYB29a in the taxol biosynthesis.

The R2R3-MYB proteins in *T. chinensis* and *A. thaliana* are classified into 36 subgroups according to their phylogenetic relationships and functions (Hu et al., 2020), of which 24 subgroups include members from *T. chinensis* and *A. thaliana*, three subgroups are specific to *T. chinensis*, while nine subgroups are specific to *A. thaliana.* The TcMYB29a, AtMYB88, and AtMYB124 were clustered in the S26 subgroup with counterparts in *A. thaliana* (Hu et al., 2020). In *A. thaliana*, AtMYB88 (FLP) and AtMYB124 proteins are extensively described as playing functions in the epidermal patterning (Lai et al., 2005), and are required for tolerating the abiotic stress (Xie et al., 2010). The loss of FLP/MYB88 function was found to make *Arabidopsis* plants more susceptible to abiotic stress, and an ABA signal may be involved in this increased sensitivity and it probably acted in the upstream of FLP/MYB88 (Xie et al., 2010). Phloem-specific TmMYB3, a recently reported R2R3-MYB TF isolated from *Taxus*, plays a role in the transcriptional regulation of taxol biosynthesis and may be important for the phloem-specific accumulation of taxol. Although both TmMYB3 and TcMYB29a function to regulate the taxol biosynthesis, they show several differences: first, they show low similarity with each other in terms of amino acid sequences (the similarity was 11.01%); second, TmMYB3 belongs to another subgroup of TcMYB29a, as TmMYB3 and TcMYB29a were separately clustered in the S4 and S26 subgroups with counterparts in *A. thaliana* (Supplementary Figure 4); third, the expression patterns of TmMYB3 and TcMYB29a were different, as TmMYB3 exhibited phloem-specific expression while TcMYB29a was highly expressed in the needles and the roots. Those results indicated that MYBs may evolve independently in the different tissues of *Taxus* to meet the need for the regulation of spatially differential taxol biosynthesis. *TcMYB29a* was highly expressed in the needles and the roots, which show a similar expression pattern with FLP/MYB88 in *Arabidopsis* plants, and its expression was highly induced after the fungal elicitor treatment based on the previous study (Cao et al., 2022). Furthermore, the PLACE website predicted that *TcMYB29a* was induced by ABA, which was verified by qRT-PCR (Figure 10 and Supplementary Figure 1). The similarities in the sequences and expression patterns between TcMYB29a and the well-characterized AtMYB88 (FLP) and AtMYB124 suggest a conserved response to ABA, although there are significant divergences between the lineages of the angiosperm and the gymnosperm. These results presented here suggest that TcMYB29a acts as a positive regulator of ABA-medicated expression of taxol biosynthesis-related genes in the *Taxus* cell cultures, and it may play its role in response to the biotic and abiotic stresses of *T. chinensis* needles and roots.

In summary, TcMYB29a was involved in the regulation of taxol biosynthesis in *T. chinensis* partly by activating the expression of the *TcT5OH* gene, and it is highly expressed in the needles and roots compared to the stem epidermis, the phloem, and the xylem. These results provided a potential explanation for the accumulation of taxol in *Taxus* needles after the fungal elicitor treatment; the expression levels of *TcMYB29a* were improved after ABA treatments but suppressed after the MeJA treatment, indicating a new ABA-mediated pathway different from the JA-mediated pathway for regulating the taxol biosynthesis. Further studies are needed to elucidate the complex signaling network of the MYB involved in the taxol biosynthesis.

## Data Availability Statement

The original contributions presented in the study are included in the article/Supplementary Material, further inquiries can be directed to the corresponding author/s.

## Author Contributions

JJ, XC, and WW planned and designed the research. WW and XC wrote the manuscript. LX, XC, LL, and WW conducted the research and analyzed the data. All authors read and approved the final manuscript.

## Conflict of Interest

The authors declare that the research was conducted in the absence of any commercial or financial relationships that could be construed as a potential conflict of interest.

## Publisher’s Note

All claims expressed in this article are solely those of the authors and do not necessarily represent those of their affiliated organizations, or those of the publisher, the editors and the reviewers. Any product that may be evaluated in this article, or claim that may be made by its manufacturer, is not guaranteed or endorsed by the publisher.

## Footnotes

1
http://web.expasy.org

2
http://www.ncbi.nlm.nih.gov/

3
http://planttfdb.gao-lab.org/tf.php?sp=Ppe&did=Prupe.I004500.1.p

4
https://www.ebi.ac.uk/Tools/hmmer

5
http://www.dna.affrc.go.jp/PLACE/

6
http://bioinformatics.psb.ugent.be/webtools/plantcare/html/

## Abbreviations

TF: transcription factor
TS: taxadiene synthase
T5OH: taxane 5a-hydroxylase
ChIP: chromatin immunoprecipitation
Y1H: yeast one-hybrid
EMSA: electrophoretic mobility shift assays
MeJA: methyl jasmonate
GGPP: geranylgeranyl diphosphate
10-DAB: 10-deacetylbaccatin III
bHLH: basic helix-loop-helix
ERF: ethylene responsive factor
DBAT: 10-deacetylbaccatin-III-10- β -O-acetyltransferase
HTH: helix-turn-helix
DAPI: 4′, 6′ -diamidino-2-phenylindole
LC-MS: liquid chromatography-mass spectrometry
MREs: MYB recognition elements
SD: synthetic dextrose
CDS: coding sequence
OE: over-expressed
DW: dry weight
qRT-PCR: quantitative real-time PCR
DBTNBT: 3′ -N-debenzoyl-2′ -deoxytaxol-N-benzoyltransferase.
