# Supplementary material for: TcMYB29a, an ABA-Responsive R2R3-MYB Transcriptional Factor, Upregulates Taxol Biosynthesis in Taxus chinensis
Source: Front Plant Sci. 2022 Mar 4;13:804593. doi: 10.3389/fpls.2022.804593 (PMC8931530; doi:10.3389/fpls.2022.804593)
Supplement: Supplementary file 1 [file Data_Sheet_1.PDF]

Supplementary Material

SupplementaryFigure

TcMYB29a-promoter

```
+ CATCTCACCA ATTTTCCCAT ACAGATGATG CTCAAAGTCA AGTCACGAGG AGGAGGATCC AATTCAAAAC
- GTAGAGTGGT TAAAAGGGTA TGTCTACTAC GAGTTTCAGT TCAGTGCTCC TCCTCCTAGG TTAAGTTTTG

+ CTCCTTTTAA CGGAATTTAC TTAAGTTCAG GCAATGTAAC AGTGAAAATG GTGCTTTTTG AGGATAAAGA
- GAGGAAAATT GCCTTAAATG AATTCAAGTC CGTTACATTG TCACTTTTAC CACGAAAAAC TCCTATTTCT

+ AAGAATGAAG GAATAGCTAC TAGGACCCAT ACTTTAGTCT GCATGCTATT ATTAAGATGT GGCTGTAAAA
- TTCTTACTTC CTTATCGATG ATCCTGGGTA TGAAATCAGA CGTACGATAA TAATTCTACA CCGACATTTT

+ GCCCACCTTT AAAGAGACAA TATTCAAACT AGGGCATAAA AAGCCTGGTA GAAAGCAGGA AATCTAATGA
- CGGGTGGAAA TTTCTCTGTT ATAAGTTTGA TCCCGTATTT TTCGGACCAT CTTTCGTCCT TTAGATTACT

+ TAATAAATTG ACAACAGTGA ATTCCTCACG TGAAACGTTA TGAAGATAAA TATGACATAG GGAAAAGTAT
- ATTATTTAAC TGTTGTCACT TAAGGAGTGC ACTTTGCAAT ACTTCTATTT ATACTGTATC CCTTTTCATA

+ GAGGGTGTTG AATACAAGGA TTAATTGATT CACTTACAAT TGACAGGTGC AGTAAAGTAA AGTAAAGATT
- CTCCCACAAC TTATGTTCCT AATTAACTAA GTGAATGTTA ACTGTCCACG TCATTTCATT TCATTTCTAA

+ AAAGATAAGG TCCTTTGTAA GTATTTTTTT GAACCAATAC ATTTGGCGCT CTTCACTATA CATAAGATTG
- TTTCTATTCC AGGAAACATT CATAAAAAAA CTTGGTTATG TAAACCGCGA GAAGTGATAT GTATTCTAAC

+ TACACTTATA CATGCATGTA CTTATATATA AAATCACTAA ATTTATGTTA ATATGAAGTC ACTAGATTTA
- ATGTGAATAT GTACGTACAT GAATATATAT TTTAGTGATT TAAATACAAT TATACTTCAG TGATCTAAAT

+ CATGAGATTG TTTGTGCAGG AATCTCGGAT GGTTTAAGAT TGGGGATAAA TTTGGATGTG GGCT ATG
- GTACTCTAAC AAACACGTCC TTAGAGCCTA CCAAATTCTA ACCCCTATTT AAACCTACAC CCGA TAC
```

start codon of *TcMYB29a*

Supplementary Figure S1 Predicated AERB site in the promoter region of *TcMYB29a.* The red box indicated the predicted cis-acting element involved in the abscisic acid responsiveness. PlantCARE (http://bioinformatics.psb.ugent.be/webtools/plantcare/html/) was used to identify the cis-acting elements of promoter of *TcMYB29a*.

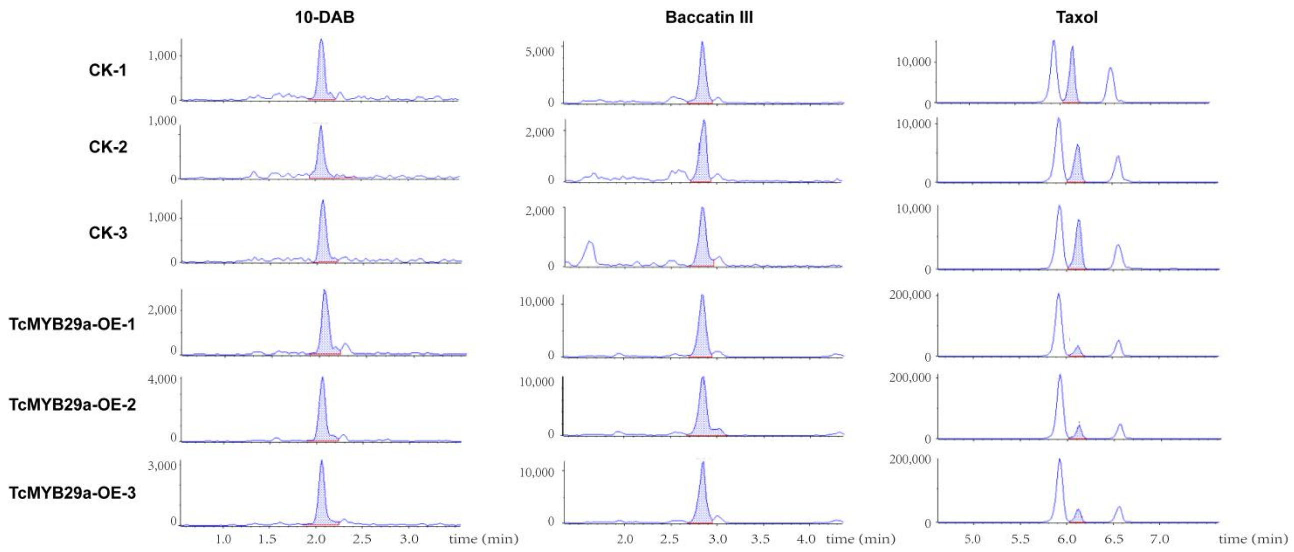

	10-DAB (μg/g DW)	Bacctain III (μg/g DW)	Taxol (μg/g DW)
CK-1	0.06	0.06	0.44
CK-2	0.05	0.03	0.23
CK-3	0.06	0.03	0.27
TcMYB29a-OE-1	0.13	0.16	1.19
TcMYB29a-OE-2	0.17	0.16	1.41
TcMYB29a-OE-3	0.13	0.15	1.32

Supplementary Figure S2 LC-MS quantified the contents of taxanes in control and TcMYB29a-OE cells.

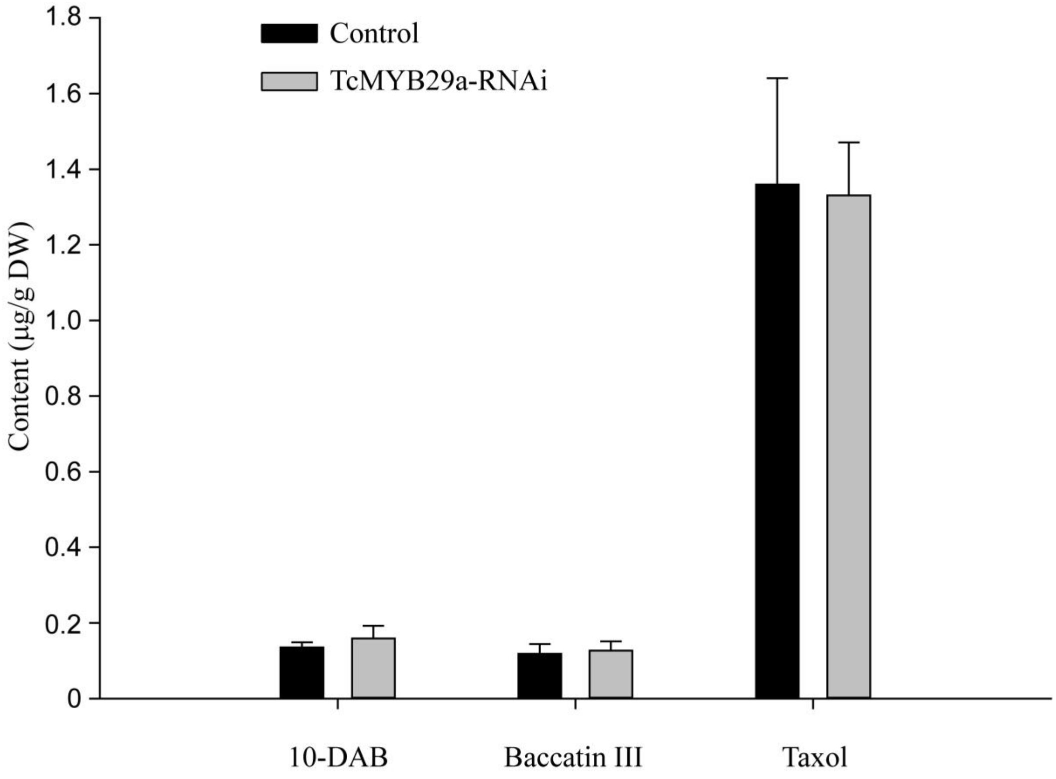

Supplementary Figure S3 LC-MS quantified the contents of taxanes in control and TcMYB29a-RNAi cells.

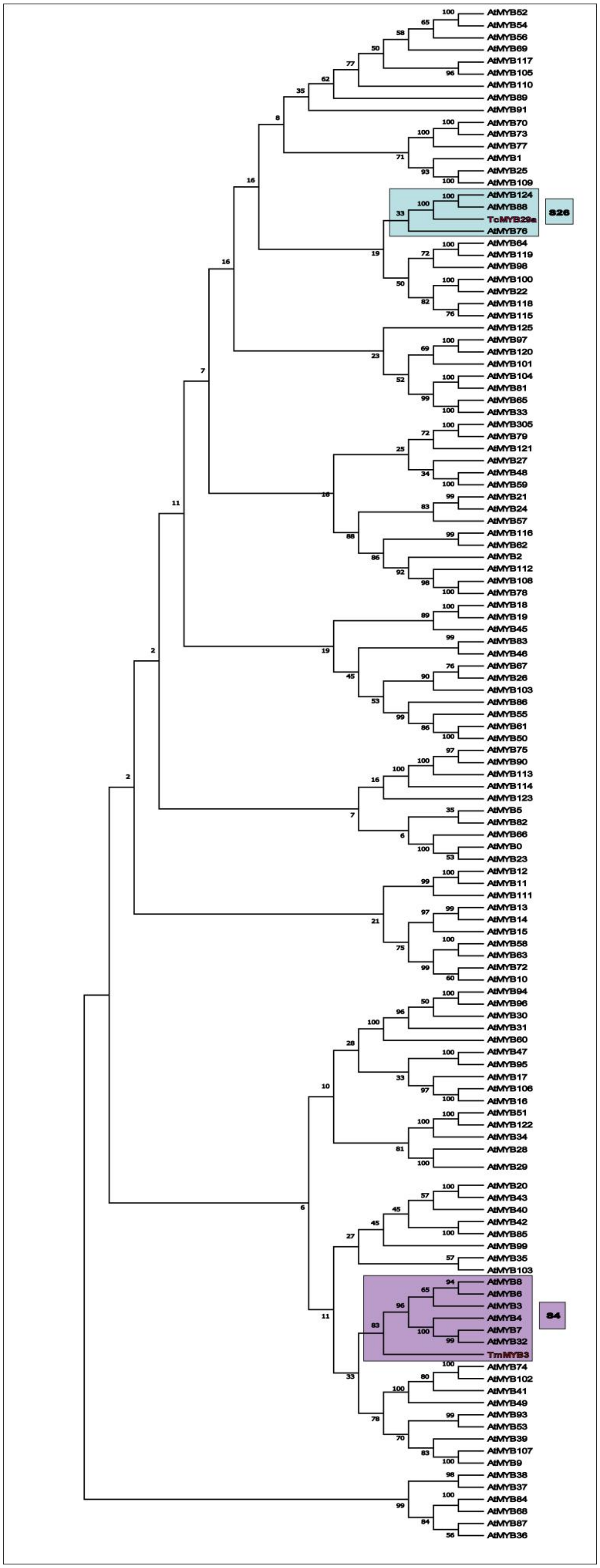

Supplementary Figure S4 Schematic representation of the relationships between TcMYB29a, TmMYB3 and the different R2R3-MYB subgroups from *Arabidopsis*. The tree was inferred using the neighbor-joining method and 1000 bootstraps with putative full length MYB amino acid sequences with Clustal W software. The subgroups were designated as previously reported (Hu *et al*., 2020)

Supplementary Table S1 Primers used in this study.

Gene Name	Sequence	Application
TcMYB29a	F: ATGAGCTATGAAGAGATGAATTCT<br>R: TTATAGCCTGTTGGATAGTGAGAG	Construction for 35s:GFP-TcMYB29a
R-1	F1: TGTAGGAGAATGGATGTCGTGAAGATT<br>R1: CCTTAATACGTGTAATTTGATGTCTCTCTTCC	CHIP-qPCR
R-2	F2: GCCTCAAGAATATGTGCATCACCCCC<br>R2: GGTGGGGAAATTGTTGGGAAGGATCG	CHIP-qPCR
R-3	F1: ACAATTCTTCTCACCCCCTGCCAT<br>R1: GGTAGCGCTAAAGGTGGGGGAA	CHIP-qPCR
R-4	F2: GTATCCATTGCATTACCCACGTGC<br>R2: CCAAGGTCATTTCAAACGTTAGATTCCAT	CHIP-qPCR
R-5	F1:TCCTTTAGTGACACCTTGCAACC<br>R1:TCCAAATGCCCAATGACCTCCTGT	CHIP-qPCR
R-6	F2:TCCCTTAGGGCTTTTTCCAGGGAC<br>R2:ACTGCCGAAAACTAATTCACAGT	CHIP-qPCR
R-7	F3:GTGTCCAATCGTCCGTAATGAGCA<br>R3:TGTAGTGAACATGGGGCGGTGA	CHIP-qPCR
R-8	F1:TGCTTTTGCGCAAGTGTGGCA<br>R1:AACCTTGCGGGCCTGATCCT	CHIP-qPCR
R-9	F2:GCCCGTGCATCACAGTGGTTGT<br>R2:AGCTAGCCGGTGCCAACTCCTT	CHIP-qPCR
CDS-1	qF: ACAAATTCGGCGGCTGCGTG<br>qR: AACGAGGCTGTCTCTGCCCCAA	CHIP-qPCR

CDS-2	qF: AGATGTCGTGGCCCGCTCAA<br>qR: AGCGCACCAGGGCCGAAAAA	CHIP-qPCR
CDS-3	qF: TGCCTGCCTTCGCCCAAAACA<br>qR: AGCCGCCCAGCAAAAGGGTA	CHIP-qPCR
CDS-4	qF: TCGATGCGGCCACCTCCAAT<br>qR:TCGCTAGCCAGGCCAATGCT	CHIP-qPCR
pY1-ABAi	Y1F: agcacatgcctcgaggtcgacAGTGGAGAATCATAGACAACTACCTCTAA<br>Y1R: gaaaagcttgaattcgagctcAGGTCGAGGAACTTGGATATAAGC	Y1H
pY2-ABAi	Y2F: agcacatgcctcgaggtcgacGTCTCAATGTATCCATTGCATTACCC<br>Y2R: gaaaagcttgaattcgagctcTTTGGAAGAAAACCAAAACAAAGA	Y1H
pB1-ABAi	B1F: gcacatgcctcgaggtcgacCCGTTACCGTTACCGTTAgagctcgaattcaagcttttc<br>B1R: gaaaagcttgaattcgagctcTAACGGTAACGGTAACGGgtcgacctcgaggcatgtgc	Y1H
pmB1-ABAi	mB1F: gcacatgcctcgaggtcgacCCGAATCCGAATCCGAATgagctcgaattcaagcttttc<br>mB1R: gaaaagcttgaattcgagctcATTCGGATTCGGATTCGGgtcgacctcgaggcatgtgc	Y1H
pY3-ABAi	Y3F: gaaaagcttgaattcgagctcTTGTCCGAAAATATTGTGTTCTTTG<br>Y3R: agcacatgcctcgaggtcgacCTCTAATTGTTCAGAACAGAGCGG	Y1H
AD-TcMYB29a	F: gccatggaggccagtgaattcATGAGCTATGAAGAGATGAATTCTATTTT<br>R: acgattcatctgcagctcgagTTATAGCCTGTTGGATAGTGAGAGCC	Y1H
B1 Probe	F: tacaatcccccgagtactccccctCCGTTAagttaagcatcctcctcttccccca<br>R: tgggggaagaggaggatccttaactTAACGGagggggggagtactcgggggattgta	EMSA
Mutant Probe	F: tacaatcccccgagtactccccctCCATagttaagcatcctcctcttccccca<br>R: tgggggaagaggaggatccttaactATGGagggggggagtactcgggggattgta	EMSA

TcT5OHpro	SP1: AGATGGCCGAGATGTTGAAGACGAGC<br>SP2: ATCTGCACCAGCTTCTCCTCGTTGGA<br>SP3: CGGTAGCGGAGGTCAAACATGGTAGT	Genome walking
TcTSpro	SP1: TGGCTGTGCCCTGTTTTCCAAACCGA<br>SP2: TCCGTCTCCGAGCGCATTGAACAT<br>SP3: ACATTGTGGTGCCACAGATCGCCA	Genome walking
TcBAPT	SP1: TTCTGAGCCGCCCAGCAAAAGGGTAA<br>SP2:TGGGAGGCCTCATATTCAACCGGAGT<br>SP3: TTCGACAATCTCCCAACTTCTCCCGC	Genome walking
TcDBTNBT	SP1:AACAGAGCACCTTCCCCCGTGCAA<br>SP2:GTGCATTTAGCGCACGCACAACAA<br>SP3: TTGGTAAAGGGCACACCCCACGTT	Genome walking
TcTS	qF: ACAAATTCGGCGGCTGCGTG<br>qR: AACGAGGCTGTCTCTGCCCCAA	qPCR
TcT5OH	qF: AGATGTCGTGGCCCGCTCAA<br>qR: AGCGCACCAGGGCCGAAAAA	qPCR
TcT7OH	qF: ACTGTTGCACGTGTCGTGGTCC<br>qR: TAGTGCGACACGCAGAACGC	qPCR
TcT10OH	qF: CGGGCCTGCGGGAAACAAAT<br>qR: ACCGAGCAAGTGCAGTGCGT	qPCR
TcBAPT	qF: TGCCTGCCTTCGCCCAAAACA<br>qR: AGCCGCCCAGCAAAAGGGTA	qPCR
TcDBTNBT	qF: TCGATGCGGCCACCTCCAAT<br>qR:TCGCTAGCCAGGCCAATGCT	qPCR
TcPAM	qF: TCGATGCGGCCACCTCCAAT<br>qR: TCGCTAGCCAGGCCAATGCT	qPCR<br>qPCR

TcT2OH	qF: AGCTGTTGAGCCGAACGGTGTC<br>qR: CGGCCCCAAAAACCCTGCAACT	qPCR
TcGAPDH	qF: CGGAGACAGTCGATCAAGC<br>qR:CCCATCCTCAACCCAATAA	qPCR
TcMYB29a	qF: GCTCAAAGGCGCAATGCAGA<br>qR: ACAGTTTGGGCAAGGAAGGCA	qPCR
35S:TcMYB29a	F: accagtctctctctcaagcttATGAGCTATGAAGAGATGAATTCTATTTT<br>R: gctcctgcagctcgaggatccTTATAGCCTGTTGGATAGTGAGAGCC	LUC
pT5OH-LUC	F: ttcctgcagcccgggggatcctAGTGGAGAATCATAGACAACTACCTCTAA<br>R: TgtttttggcgtcttccatggTTTGGAAGAAAACCAAAACAAAGA	LUC
pmT5OH-LUC	F: ttcctgcagcccgggggatcctAGTGGAGAATCATAGACAACTACCTCTAA<br>FP-R: tgggggaagaggaggatccttaactATGGagggggggagtactcgggggattgta<br>FP-F: tacaatcccccgagtactccccccctCCATagttaagcatcctcctcttcccca<br>R: TgtttttggcgtcttccatggTTTGGAAGAAAACCAAAACAAAGA	LUC

Reference

Hu, X., Zhang, L., Wilson, I., Shao, F., and Qiu, D.. (2020). The R2R3-MYB transcription factor family in *Taxus chinensis*: identification, characterization, expression profiling and posttranscriptional regulation analysis. Peer J. 8, e8473. doi:10.7717/peerj.8473.
