# Supplementary material for: TcMYB29a, an ABA-Responsive R2R3-MYB Transcriptional Factor, Upregulates Taxol Biosynthesis in Taxus chinensis
Source: Front Plant Sci. 2022 Mar 4;13:804593. doi: 10.3389/fpls.2022.804593 (PMC8931530; doi:10.3389/fpls.2022.804593)
Supplement: Supplementary file 2 [file Data_Sheet_2.docx]

Raw data: <https://www.jianguoyun.com/p/DYVlFMEQteb9CRi53ZcE>
